# Effectiveness of 2-Dose BNT162b2 (Pfizer BioNTech) mRNA Vaccine in Preventing SARS-CoV-2 Infection Among Children Aged 5–11 Years and Adolescents Aged 12–15 Years — PROTECT Cohort, July 2021–February 2022

**DOI:** 10.15585/mmwr.mm7111e1

**Published:** 2022-03-18

**Authors:** Ashley L. Fowlkes, Sarang K. Yoon, Karen Lutrick, Lisa Gwynn, Joy Burns, Lauren Grant, Andrew L. Phillips, Katherine Ellingson, Maria V. Ferraris, Lindsay B. LeClair, Clare Mathenge, Young M. Yoo, Matthew S. Thiese, Lynn B. Gerald, Natasha Schaefer Solle, Zuha Jeddy, Leah Odame-Bamfo, Josephine Mak, Kurt T. Hegmann, Joe K. Gerald, Jezahel S. Ochoa, Mark Berry, Spencer Rose, Julie Mayo Lamberte, Purnima Madhivanan, Felipe A. Pubillones, Ramona P. Rai, Kayan Dunnigan, John T. Jones, Karl Krupp, Laura J. Edwards, Edward J. Bedrick, Brian E. Sokol, Ashley Lowe, Hilary McLeland-Wieser, Krystal S. Jovel, Deanna E. Fleary, Sana M. Khan, Brandon Poe, James Hollister, Joanna Lopez, Patrick Rivers, Shawn Beitel, Harmony L. Tyner, Allison L. Naleway, Lauren E.W. Olsho, Alberto J. Caban-Martinez, Jefferey L. Burgess, Mark G. Thompson, Manjusha Gaglani

**Affiliations:** ^1^CDC COVID-19 Emergency Response Team; ^2^Rocky Mountain Center for Occupational and Environmental Health, Department of Family and Preventive Medicine, University of Utah Health, Salt Lake City, Utah; ^3^College of Medicine - Tucson, University of Arizona, Tucson, Arizona; ^4^Leonard M. Miller School of Medicine, University of Miami, Miami, Florida; ^5^Abt Associates, Rockville, Maryland; ^6^Mel and Enid Zuckerman College of Public Health, University of Arizona, Tucson, Arizona; ^7^Baylor Scott & White Health, Texas, Temple, Texas; ^8^St. Luke’s Regional Health Care System, Duluth, Minnesota; ^9^Kaiser Permanente Northwest Center for Health Research, Portland, Oregon; ^10^Texas A&M University College of Medicine, Temple, Texas.

The BNT162b2 (Pfizer-BioNTech) mRNA COVID-19 vaccine was recommended by CDC’s Advisory Committee on Immunization Practices for persons aged 12–15 years (referred to as adolescents in this report) on May 12, 2021, and for children aged 5–11 years on November 2, 2021 (*1*–*4*). Real-world data on vaccine effectiveness (VE) in these age groups are needed, especially because when the B.1.1.529 (Omicron) variant became predominant in the United States in December 2021, early investigations of VE demonstrated a decline in protection against symptomatic infection for adolescents aged 12–15 years and adults* (*5*). The PROTECT^†^ prospective cohort of 1,364 children and adolescents aged 5–15 years was tested weekly for SARS-CoV-2, irrespective of symptoms, and upon COVID-19–associated illness during July 25, 2021–February 12, 2022. Among unvaccinated participants (i.e., those who had received no COVID-19 vaccine doses) with any laboratory-confirmed SARS-CoV-2 infection, those with B.1.617.2 (Delta) variant infections were more likely to report COVID-19 symptoms (66%) than were those with Omicron infections (49%). Among fully vaccinated children aged 5–11 years, VE against any symptomatic and asymptomatic Omicron infection 14–82 days (the longest interval after dose 2 in this age group) after receipt of dose 2 of the Pfizer-BioNTech vaccine was 31% (95% CI = 9%–48%), adjusted for sociodemographic characteristics, health information, frequency of social contact, mask use, location, and local virus circulation. Among adolescents aged 12–15 years, adjusted VE 14–149 days after dose 2 was 87% (95% CI = 49%–97%) against symptomatic and asymptomatic Delta infection and 59% (95% CI = 22%–79%) against Omicron infection. Fully vaccinated participants with Omicron infection spent an average of one half day less sick in bed than did unvaccinated participants with Omicron infection. All eligible children and adolescents should remain up to date with recommended COVID-19 vaccinations.

PROTECT is a prospective cohort study monitoring SARS-CoV-2 infections among participants aged 6 months–17 years in jurisdictions in four states (Arizona, Florida, Texas, and Utah), initiated in July 2021 (*6*). Upon enrollment, parents or legal guardians provided the participants’ demographic, health, vaccination history, and prior SARS-CoV-2 infection information; the number of hours and percentage of time participants wore masks in school and in the community were reported monthly.^§^ Vaccination was verified by vaccine cards, electronic medical records, and state immunization registries. Active surveillance for SARS-CoV-2 infection and any COVID-19–associated symptoms^¶^ within the preceding 7 days occurred through weekly submission of a survey and nasal swab for reverse transcription–polymerase chain reaction testing and viral whole genome sequencing.** Specific symptoms and duration, hours of school missed because of illness, and receipt of medical care were documented through the electronic surveys.

For the calculation of VE, person-time for adolescents aged 12–15 years began at the start of active surveillance on July 25, 2021, and ended February 12, 2022, or, for adolescents eligible for a third (booster) dose (≥5 months after second mRNA vaccine dose receipt), person-time ended when a booster dose was authorized on January 3, 2022.^††^ For children aged 5–11 years, person-time for Omicron models began 6 weeks after the Pfizer-BioNTech vaccine was recommended on November 2, 2021, and ended February 12, 2022. COVID-19 characteristics and comparisons between Delta and Omicron infections were assessed. Cox proportional hazards models with time-varying vaccination status were used to calculate hazard ratios of unvaccinated to vaccinated participants with no prior SARS-CoV-2 infection (≥14 days after receipt of a second Pfizer-BioNTech vaccine dose), weighted for inverse probability of vaccination using sociodemographic characteristics, health information, frequency of social contact, mask use, location, and local virus circulation. Characteristics of Omicron infections among vaccinated and unvaccinated participants were also compared.^§§^ All analyses were conducted using SAS software (version 9.4; SAS Institute) or R software (version 4.1.2; R Foundation). This study was reviewed by CDC and approved by the institutional review boards at participating sites or under a reliance agreement with Abt Associates institutional review board and was conducted consistent with applicable federal law and CDC policy.^¶¶^

The study sample comprised 1,364 participants, including 1,052 (77%) children aged 5–11 years and 312 (23%) adolescents aged 12–15 years (Table 1).*** Overall, 76% of participants lived in Arizona, 52% were female, 76% were White, 34% were Hispanic, and 10% had at least one chronic medical condition. Of 381 SARS-CoV-2 infections among children aged 5–11 years, and 127 infections among adolescents aged 12–15 years, 352 (93%) and 97 (76%), respectively, were Omicron infections.

**TABLE 1:** Characteristics of children and adolescents aged 5–15 years in the PROTECT* Pfizer-BioNTech COVID-19 vaccine effectiveness cohort — four states, July 2021–February 2022

Characteristic	All participants, no. (column %)	COVID-19 vaccination status, no. (row %)		P-value^§^	All SARS-CoV-2 infections, no. (row %)		P-value^§^
		Unvaccinated	≥1 dose^†^		Yes^¶^	No	
**All participants**	**1,364**	**386 (28.3)**	**978 (71.7)**	**—**	**508 (37.2)**	**856 (62.8)**	**—**
**Geographic location**							
Phoenix, Arizona	232 (17.0)	53 (22.8)	179 (77.2)	<0.001	87 (37.5)	145 (62.5)	<0.001
Tucson, Arizona	682 (50.0)	127 (18.6)	555 (81.4)		214 (31.4)	468 (68.6)	
Other areas in Arizona	121 (8.9)	50 (41.3)	71 (58.7)		55 (45.5)	66 (54.5)	
Miami, Florida	114 (8.4)	59 (51.8)	55 (48.2)		50 (43.9)	64 (56.1)	
Temple, Texas	84 (6.2)	41 (48.8)	43 (51.2)		47 (56.0)	37 (44.0)	
Salt Lake City, Utah	131 (9.6)	56 (42.7)	75 (57.3)		55 (42.0)	76 (58.0)	
**Age group, yrs**							
5–11	1,052 (77.1)	301 (28.6)	751 (71.4)	0.637	381 (36.2)	671 (63.8)	0.150
12–15	312 (22.9)	85 (27.2)	227 (72.8)		127 (40.7)	185 (59.3)	
**Sex**							
Female	713 (52.3)	203 (28.5)	510 (71.5)	0.883	254 (35.6)	459 (64.4)	0.196
Male	651 (47.7)	183 (28.1)	468 (71.9)		254 (39.0)	397 (61.0)	
**Ethnicity (all races)**							
Hispanic	469 (34.4)	158 (33.7)	311 (66.3)	0.264	163 (34.8)	306 (65.2)	0.312
Non-Hispanic	895 (65.6)	228 (25.5)	667 (74.5)		345 (38.5)	550 (61.5)	
Race (all ethnicities)**							
White	1,032 (75.7)	284 (27.5)	748 (72.5)	0.260	392 (38.0)	640 (62.0)	0.318
Other races	332 (24.3)	102 (30.7)	230 (69.3)		116 (34.9)	216 (65.1)	
**No. of children in household**							
1	204 (15.0)	52 (25.5)	152 (74.5)	0.334	66 (32.4)	138 (67.6)	0.117
≥2	1160 (85.0)	334 (28.8)	826 (71.2)		442 (38.1)	718 (61.9)	
**Chronic condition^††^**							
One or more	139 (10.2)	39 (28.1)	100 (71.9)	0.835	57 (41.0)	82 (59.0)	0.718
None	1,225 (89.8)	347 (28.3)	878 (71.7)		451 (36.8)	774 (63.2)	
**Daily medication^§§^**							
None	823 (60.3)	194 (50.3)	629 (64.3)	0.121	287 (56.5)	536 (62.6)	0.626
1	116 (8.5)	21 (5.4)	95 (9.7)		40 (7.9)	76 (8.9)	
2	52 (3.8)	5 (1.3)	47 (4.8)		21 (4.1)	31 (3.6)	
3	24 (1.8)	4 (1.0)	20 (2.0)		9 (1.8)	15 (1.8)	
≥4	16 (1.2)	4 (1.0)	12 (1.2)		3 (0.6)	13 (1.5	
**Insurance**							
Private	1,052 (77.1)	247 (23.5)	805 (76.5)	<0.001	385 (36.6)	667 (63.4)	0.203
Public	197 (14.4)	78 (39.6)	119 (60.4)		84 (42.6)	113 (57.4)	
None or did not respond	115 (8.4)	61 (53.0)	54 (47.0)		39 (33.9)	76 (66.1)	
**Average weekly social contact and mask use^¶¶^**							
Hours attending school, mean (SE)	37.9 (0.2)	36.1 (0.4)	38.5 (0.2)	<0.001	36.8 (0.3)	38.6 (0.2)	0.230
Percentage of school time masked, mean (SE)	78.0 (0.2)	59.9 (0.5)	83.8 (0.2)	<0.001	71.3 (0.4)	81.8 (0.2)	<0.001
Hours in community, mean (SE)	10.7 (0.1)	11.6 (0.2)	10.4 (0.1)	0.157	11.6 (0.1)	10.1 (0.1)	0.041
Percentage of community time masked, mean (SE)	64.3 (0.2)	47.6 (0.5)	69.6 (0.2)	<0.001	57.5 (0.4)	68.1 (0.3)	<0.001
Hours of COVID-19 exposure, mean (SE)	2.1 (0.1)	2.8 (0.2)	1.8 (0.1)	0.389	2.7 (0.1)	1.7 (0.1)	<0.001

* PROTECT (Pediatric Research Observing Trends and Exposures in COVID-19 Timelines) is conducted in Phoenix and Tucson, Arizona; Miami, Florida; Temple, Texas; and Salt Lake City, Utah.

^†^ COVID-19 vaccination status excludes participants with reverse transcription–polymerase chain reaction–confirmed SARS-CoV-2 infection during the first 13 days after receiving their first vaccine dose (n = 36).

^§^ P-values comparing the percentage of persons vaccinated with those not vaccinated and those with SARS-CoV-2 infections with those not infected by sociodemographic and health categories were calculated using Pearson’s chi-square test. P-values for continuous variables were calculated using the Kruskal-Wallis test.

^¶^ SARS-CoV-2 infections were detected by reverse transcription–polymerase chain reaction testing.

** Among 332 children of other races, 111 (33.4%) identified as multiracial, 43 (13.0%) as Asian, 28 (8%) as Black or African American, eight (2%) as American Indian or Alaskan Native, three (1%) as Native Hawaiian or other Pacific Islander, and 14 (4%) as other; race was missing, or respondent declined to answer for 125 (38%).

^††^ Chronic conditions included asthma or chronic lung disease, cancer, diabetes, heart disease, hypertension, immunosuppression or autoimmune disorder, kidney disease, liver disease, neurologic or neuromuscular disorder, or other chronic conditions.

**^§§^** Number of daily medications prescribed by a physician were reported by participant parent or legal guardian at study enrollment.

^¶¶^ Participants were asked to respond to monthly survey questions about COVID-19 exposure, social contact, and mask use during the previous 7 days. The average of monthly responses is calculated for each person. Average values across persons were compared according to their vaccination and SARS-CoV-2 infection status at the time of this analysis. School hours represent in-person school, child care, or before- or after-school care attendance.

Participants who received ≥1 doses of vaccine were reported to have worn a mask during 84% of school hours and 70% of hours in the community, whereas unvaccinated children were masked during 60% of school hours and 48% of hours in the community (p <0.001 for both). Lower percentages of masked time in school (71%) and in the community (58%) were reported for participants with SARS-CoV-2 infection, compared with those of participants who had no infection (82% and 68%, respectively) (p <0.001).

Among 252 unvaccinated participants with SARS-CoV-2 infections throughout the study period, 112 (44%) were asymptomatic; unvaccinated participants with Omicron infections were less likely to report COVID-19 symptoms (49%) than were those with Delta infections (66%) (crude odds ratio = 0.5; 95% CI = 0.3–0.8) (Table 2). Overall, unvaccinated participants with COVID-19 symptoms experienced an average of 6.9 days with illness symptoms, spent an average of 1.9 days sick in bed, and missed an average of 24.0 hours of school because of illness. Omicron-associated COVID-19 symptoms lasted an average of 5.3 days and resulted in an average of 18.8 hours of missed school, which was 3.4 fewer days of symptoms (95% CI = –5.7 to –1.0) and 10.6 fewer hours of school missed (95% CI = –18.6 to –2.7) than Delta-associated COVID-19.

**TABLE 2:** Comparison of SARS-CoV-2 Delta and Omicron variant infection characteristics among unvaccinated children and adolescents aged 5–15 years and by Pfizer-BioNTech vaccination status among Omicron infections — PROTECT* cohort study, four states, July 2021–February 2022

Characteristic	Participant vaccination status at time of infection							
	Unvaccinated					2 COVID-19 vaccine doses received 14–149 days before infection		
	Infections, no. (%)			OR or mean difference, Omicron versus Delta (95% CI)^§^	P-value^§^	Omicron No. (%)^¶^	Adjusted OR or mean difference, vaccinated versus unvaccinated (95% CI)**	P-value**
	Total^†^	Delta	Omicron					
Total participants, no. (%)	**252 (100)**	102 (100)	150 (100.0)	—	—	186 (100.0)	—	—
COVID-19–associated symptoms, no. (%)^††^	**140 (55.6)**	67 (65.7)	73 (48.7)	2.0 (1.20 to 3.45)	0.008	116 (62.4)	0.91 (0.48 to 1.59)	0.669
Febrile symptoms, no. (%)**^§§^**	**88 (62.9)**	38 (56.7)	50 (68.5)	1.7 (0.83 to 3.31)	0.151	66 (56.9)	0.48 (0.23 to 1.03)	0.062
Received medical care, no. (%)	**23 (16.4)**	11 (16.4)	12 (16.4)	1.0 (0.41 to 2.45)	0.997	18 (15.5)	1.0 (0.43 to 2.48)	0.949
Total days of symptoms, mean (SE)	**6.9 (6.7)**	8.6 (8.0)	5.3 (5.4)	–3.4 (–5.7 to –1.0)	0.006	6.3 (3.9)	0.8 (–1.8 to 2.7)	0.426
Days spent sick in bed, mean (SE)	**1.9 (2.4)**	1.7 (2.7)	2.1 (2.1)	0.4 (–0.4 to 1.2)	0.322	1.4 (1.6)	–0.6 (–1.1 to –0.1)	0.016
Hours of missed school, mean (SE)	**24.0 (23.5)**	29.5 (24.1)	18.8 (21.8)	–10.6 (–18.6 to –2.7)	0.010	26.2 (17.5)	11.1 (4.6 to 17.6)	0.010

**Abbreviation:** OR = odds ratio.

* PROTECT (Pediatric Research Observing Trends and Exposures in COVID-19 Timelines) is conducted in Phoenix and Tucson, Arizona; Miami, Florida; Temple, Texas; and Salt Lake City, Utah.

^†^ Includes all participants aged 5–15 years, and infections that occurred at any time during the cohort study (July 25, 2021–February 12, 2022). However, of 275 total infections among unvaccinated participants, only 252 completed a post-illness survey capturing symptoms.

^§^ Severity of infection, comparing Delta infections as the referent group with Omicron infections, was assessed by variant type among unvaccinated children and adolescents. Logistic and linear regression models were used for dichotomous and continuous outcome measures, respectively. P-values <0.05 were considered statistically significant.

^¶^ Of 198 total infections in persons that occurred 14–149 days after dose 2 receipt, 186 completed a post-illness survey to report symptoms. This excludes four Omicron infections in persons aged 12–15 years with infection ≥150 days after receipt of dose 2.

** Severity of infection was assessed by vaccination status, comparing unvaccinated children as the referent group with children vaccinated 14–149 days earlier, among Omicron infections. Comparison of vaccinated and unvaccinated participants with Delta infections was not included because of the limited number of vaccinated children with Delta infections. Logistic and linear regression models were used for dichotomous and continuous outcome measures, respectively, weighted for inverse probability of vaccination by site, sociodemographic characteristics, health information, and knowledge, attitudes, and practices regarding SARS-CoV-2 infection and vaccine.

^††^ COVID-19–associated illness signs and symptoms included fever >100°F (37.8°C), chills, cough, shortness of breath, sore throat, diarrhea, muscle or body aches, change in smell or taste; runny nose, fatigue or being run-down, decreased activity, and irritability or crankiness were also included for nonverbal children.

**^§§^** Febrile symptoms were defined as symptoms of feverishness or chills, or a measured temperature >100.4°F (38°C).

Among the 1,052 participants aged 5–11 years, 682 (65%) received 2 vaccine doses, 69 (7%) received 1 dose, and 301 (29%) were unvaccinated. Adjusted VE against symptomatic and asymptomatic Omicron infection 14–82 days after receipt of dose 2 (the longest interval after dose 2 in this age group) was 31% (95% CI = 9%–48%) (Table 3).

**TABLE 3:** COVID-19 Pfizer-BioNTech vaccine effectiveness against asymptomatic or symptomatic SARS-CoV-2 infection among children and adolescents aged 5–15 years, by time since receipt of second vaccine dose and variant — PROTECT* cohort study, four states, July 2021–February 2022

Age group and COVID-19 vaccination status (no. of days since receipt of most recent dose)	No. of contributing participants^†^	Total person-days	Median no. of days (IQR)	No. of SARS-CoV-2 infections^§^	VE, % (95% CI)	
					Unadjusted	Adjusted^¶^
**Children aged 5–11 yrs**						
**Omicron variant infections**						
Unvaccinated (referent)	336	13,801	41 (28 to 62)	137	—	—
2 doses (14–82 days)	640	29,996	53 (34 to 61)	184	47 (32 to 59)	31 (9 to 48)
**Adolescents aged 12–15 yrs**						
**Delta variant infections**						
Unvaccinated (referent)	139	9,786	65 (25 to 107)	23	—	—
2 doses (≥14 days)	193	23,575	142 (91 to 156)	7	87 (70 to 95)	81 (51 to 93)
2 doses (14–149 days)	188	16,517	97 (75 to 105)	3	93 (76 to 98)	87 (49 to 97)
2 doses (≥150 days)	138	7,058	57 (49 to 63)	4	67 (0 to 89)	60 (−35 to 88)
**Omicron variant infections**						
Unvaccinated (referent)	76	3,001	37 (24 to 62)	38	—	—
2 doses (≥14 days)	192	5,432	22 (22 to 31)	18	64 (37 to 80)	59 (24 to 78)
2 doses (14–149 days)	65	2,623	42 (28 to 56)	14	62 (30 to 79)	59 (22 to 79)
2 doses (≥150 days)	134	2,809	22 (22 to 22)	4	74 (16 to 92)	62 (−28 to 89)

**Abbreviations:** SMD = standard mean difference; VE = vaccine effectiveness.

* PROTECT (Pediatric Research Observing Trends and Exposures in COVID-19 Timelines) is conducted in Phoenix and Tucson, Arizona; Miami, Florida; Temple, Texas; and Salt Lake City, Utah.

^†^ Vaccination status varied with time, therefore, contributing participants in vaccination categories do not equal the number of participants in the study because participants could contribute to more than one vaccination category.

^§^ Of 275 SARS-CoV-2 infections among unvaccinated participants, 98 occurred among children aged 5–11 years either before vaccine availability (n = 60) or were Delta infections (n = 17) for whom VE was not calculated. Among vaccinated participants, 61 occurred after receipt of dose 1 and <14 days after dose 2; two children aged 5–11 years were vaccinated before authorization, and two had Delta infections among children aged 5–11 years for whom VE was not calculated.

^¶^ Adjusted VE is inversely weighted for propensity to be vaccinated. Among children aged 5–11 years, all covariates met balance criteria of SMD <0.2 after weighting for the Delta variant model. For the Omicron variant model, all covariates met balance criteria of SMD <0.2 after weighting, except local virus circulation and social (school or community) mask use, which both changed the VE estimate by ≥5% when added to the model, and thus remained in the final model as covariates. Among adolescents aged 12–15 years, all covariates met balance criteria of SMD <0.2 after weighting except social mask use, which also changed the VE estimate by ≥5% when added to the Delta variant model, and thus remained in the final model as a covariate. For the Omicron variant model, all covariates met balance criteria of SMD <0.2 after weighting, except local virus circulation, social (school or community) mask use, and number of medications. Only local virus circulation changed the VE estimate by ≥5% when added to the model, and thus remained in the final model as a covariate.

Among 312 adolescents aged 12–15 years, 212 (68%) received 2 vaccine doses, 15 (5%) received 1 dose, and 85 (27%) were unvaccinated. The adjusted VE at 14–149 days after receipt of dose 2 was 87% (95% CI = 49%–97%) against Delta infection and 59% (95% CI = 22%–79%) against Omicron infection. Adjusted VE ≥150 days after dose 2 was 60% against Delta infection and 62% against Omicron, with wide CIs that included zero.

Among 186 vaccinated participants with Omicron infections (174 [93%] in children aged 5–11 years and 13 [7%] in adolescents aged 12–15 years), 37.6% were asymptomatic; those reporting COVID-19 symptoms spent 1.4 days in bed, which was 0.6 days fewer than reported for unvaccinated participants (95% CI = –1.1 to –0.1) (Table 2), after adjusting for the propensity to be vaccinated. Conversely, vaccinated participants with Omicron infections stayed home from school 26.2 hours, an adjusted mean of 11 hours more than that reported for unvaccinated participants (95% CI = 4.6–17.6). Overall, medical care–seeking was reported for 16.4% of unvaccinated participants with Omicron infections and 15.5% of vaccinated participants, which was not significantly different.

## Discussion

In this prospective cohort study of children and adolescents aged 5–15 years that included routine weekly SARS-CoV-2 testing, irrespective of symptoms, 2 doses of Pfizer-BioNTech vaccines were effective in preventing symptomatic and asymptomatic SARS-CoV-2 infections, although effectiveness varied by variant. VE point estimates were highest against Delta variant infections among adolescents aged 12–15 years and lowest against Omicron variant infections among children aged 5–11 years.

The SARS-CoV-2 infections prevented by vaccination differed by variant. Approximately one half (51%) of all Omicron infections were asymptomatic compared with approximately one third (34%) of Delta infections. However, when children or adolescents experienced symptomatic COVID-19, the illnesses disrupted life at home and school; on average COVID-19 lasted 7 days, two of which were spent sick in bed, and resulted in 24 hours of missed school.

Two doses of Pfizer-BioNTech vaccine received <5 months earlier were moderately effective (31%) in preventing symptomatic and asymptomatic Omicron infection among children aged 5–11 years and 59% effective among adolescents aged 12–15 years. The wide and overlapping CIs indicate that these age-specific VE point estimates might not be significantly different and are similar to a recent report of VE of 45%–51% for 2 doses, received within 150 days, against Omicron COVID-19–associated emergency department and urgent care visits among children and adolescents aged 5–15 years (*7*). Participants who were infected with Omicron despite receipt of 2 vaccine doses spent an average of one half day less sick in bed than did unvaccinated participants with Omicron infections. Also, similar to studies of children (*7*) and adults (*6*), among adolescents aged 12–15 years, point estimates for VE of 2 doses received within the previous 150 days were lower against Omicron than Delta infections, although these differences were not statistically significant.

The findings in this report are subject to at least five limitations. First, despite the use of robust adjusted models previously applied in other cohort studies (*8*), VE estimates might have been biased by residual confounding due to other differences between vaccinated and unvaccinated participants. For example, vaccinated participants reported wearing face masks significantly more often at school and in the community than did unvaccinated participants. Second, although PROTECT is among the largest studies with routine weekly SARS-CoV-2 testing, the relatively small number of infections within vaccination categories among certain age groups reduced precision of VE estimates. Estimates of VE at ≥150 days after dose 2 had very wide CIs, and thus it is unclear whether VE wanes with increased time since vaccination. Third, data were not available to assess possible reasons that vaccinated participants with COVID-19 might have missed more school than did unvaccinated participants despite unvaccinated participants reporting more days sick in bed. Fourth, these interim estimates do not include separate analyses of VE against asymptomatic infection and symptomatic infection at this time. Finally, although this study was conducted in multiple sites and included more than 1,300 participants, findings from the study sample might not be generalizable to all populations.

This study provides evidence that receipt of 2 doses of Pfizer-BioNTech vaccine is effective in preventing both asymptomatic and symptomatic SARS-CoV-2 infection with the Omicron variant among children and adolescents aged 5–15 years. All eligible children and adolescents should remain up to date with recommended COVID-19 vaccinations.

**Summary**

**What is already known about this topic?**

Receipt of 2 doses of Pfizer-BioNTech COVID-19 vaccine has been shown to be effective in preventing infection with the SARS-CoV-2 B.1.617.2 (Delta) variant in persons aged ≥12 years.

**What is added by this report?**

Children and adolescents aged 5–15 years were tested for SARS-CoV-2 weekly, irrespective of symptoms, during July 2021–February 2022. Approximately one half of Omicron infections in unvaccinated children and adolescents were asymptomatic. Two doses of Pfizer-BioNTech COVID-19 vaccine reduced the risk of Omicron infection by 31% among children aged 5–11 years and by 59% among persons aged 12–15 years.

**What are the implications for public health practice?**

All eligible children and adolescents should remain up to date with recommended COVID-19 vaccinations.

## Footnotes

*
https://www.medrxiv.org/content/10.1101/2021.12.10.21267408v3

† PROTECT (Pediatric Research Observing Trends and Exposures in COVID-19 Timelines) is conducted in Phoenix and Tucson, Arizona; Miami, Florida; Temple, Texas; and Salt Lake City, Utah.

§ Parents or legal guardians were asked, “In the past 7 days, how many hours did [participant] spend in the community, meaning outside the home and NOT at school, daycare, or before-/after-school care? (For example: in stores, at parks, at work, playing sports, or at summer camp)” followed by, “In the past 7 days when [participant] was in the community, for what % of time did they wear a face mask?”

¶ COVID-19–associated illness signs and symptom included fever >100°F (37.8°C), chills, cough, shortness of breath, sore throat, diarrhea, muscle or body aches, change in smell or taste, runny nose, fatigue or being run-down, decreased activity, and irritability or crankiness were also included for nonverbal children. A short survey submitted with the specimen asked the parent or guardian if the child had any COVID-19 symptoms in the previous 7 days.

** Specimens not eligible (cycle threshold [Ct] value >30) for sequencing were assumed to contain the Delta variant from July 25, 2021, to the date when the Omicron variant accounted for >50% of sequenced viruses at each study site. During weeks of Omicron and Delta cocirculation, 62% (38 of 61) of SARS-CoV-2 samples were sequenced. Point estimates of VE changed <5% when unsequenced samples were removed; however, 95% CIs were wider because of decreased sample size and precision.

†† The Food and Drug Administration (FDA) amended the Emergency Use Authorization (EUA) for the Pfizer-BioNTech vaccine to include adolescents aged 12–15 years on May 10, 2021 (https://www.fda.gov/news-events/press-announcements/coronavirus-covid-19-update-fda-authorizes-pfizer-biontech-covid-19-vaccine-emergency-use), and CDC recommended the Pfizer-BioNTech vaccine for this age group on May 12, 2021 (https://www.cdc.gov/media/ releases/2021/s0512-advisory-committee-signing.html). FDA amended EUA for the Pfizer-BioNTech COVID-19 vaccine to expand the use of a single booster dose to include use in persons aged 12–15 years, 5 months after receipt of the second primary series mRNA COVID-19 vaccine dose on January 3, 2022, and CDC recommended a third dose for this age group on January 5, 2022 (https://www.cdc.gov/media/releases/2022/s0105-Booster-Shot.html). FDA authorized EUA for the Pfizer-BioNTech vaccine for children aged 5–11 years on October 29, 2021 (https://www.fda.gov/news-events/press-announcements/fda-authorizes-pfizer-biontech-covid-19-vaccine-emergency-use-children-5-through-11-years-age), and CDC recommended the Pfizer-BioNTech vaccine for this age group on November 2, 2021 (https://www.cdc.gov/media/releases/2021/s1102-PediatricCOVID-19Vaccine.html).

§§ Severity of infection was assessed by variant type among unvaccinated children and by vaccination status among Omicron infections because of limited number of Delta infections among vaccinated persons. Logistic and linear regression models were used for dichotomous and continuous outcome measures, respectively, weighted for inverse probability of vaccination by site, sociodemographic characteristics, health information, including number of chronic medical conditions, number of daily prescription medications, and influenza vaccination history, and SARS-CoV-2 infection and vaccine knowledge, attitudes, and practices. For VE and severity of infection models, any variable that was unbalanced (standardized mean difference ≥0.2) after weighting and that modified the model outcome point estimate ≥5%, was added to the model as a covariate. Participants with partial vaccination or <14 days after second dose were excluded from VE and attenuation analyses.

¶¶ 45 C.F.R. part 46, 21 C.F.R. part 56, 42 U.S.C. Sect. 241(d), 5 U.S.C. Sect. 552a, 44 U.S.C. Sect. 3501 et seq.

*** The study excluded 167 children and adolescents aged 5–15 years with documented SARS-CoV-2 infection before enrollment or start of follow-up, 90 who failed to complete weekly nasal swabs or were not in surveillance during the variant-specific follow-up period, and 17 who received a vaccine product other than Pfizer-BioNTech or had incomplete vaccination information.
